# Clinical Variability Within the *PLOD2*-Associated Phenotypic Continuum: Three Novel Variants in Four Patients from a Descriptive Case Series

**DOI:** 10.3390/genes17050556

**Published:** 2026-05-05

**Authors:** Elena S. Merkuryeva, Evgeniya A. Melnik, Vladimir M. Kenis, Svetlana I. Trofimova, Olga E. Agranovich, Yuri V. Buklemishev, Khushnud K. Rustamov, Denis V. Chistol, Tatiana S. Nagornova, Viktoriia V. Zabnenkova, Tatiana V. Markova

**Affiliations:** 1 Research Centre for Medical Genetics, 115522 Moscow, Russia; 2 The Turner Scientific Research Institute for Children’s Orthopedics, 196603 Saint Petersburg, Russia; 3 The National Medical Research Center of Traumatology and Orthopedics Named after N.N. Priorov, 127299 Moscow, Russia; 4 Republican Specialized Scientific and Practical Center for Traumatology and Orthopedics, Tashkent 100047, Uzbekistan; 5 Tashkent Research Center for Medical Genetics, Tashkent 100060, Uzbekistan

**Keywords:** Bruck syndrome, osteogenesis imperfecta, *PLOD2*, lysyl hydroxylase 2, collagen, skeletal dysplasia, contractures, arthrogryposis

## Abstract

**Background/Objectives**: Bruck syndrome type 2 (BS2) is an ultra-rare autosomal recessive disorder within the osteogenesis imperfecta (OI) spectrum caused by biallelic pathogenic variants in *PLOD2*, which encodes lysyl hydroxylase 2 (LH2), an enzyme essential for bone-specific collagen cross-linking. Marked clinical heterogeneity complicates diagnosis, particularly in patients with atypical or incomplete presentations. We aimed to further delineate the clinical and molecular spectrum of *PLOD2*-associated disease. **Methods**: In this descriptive case series, we performed clinical, radiological, and molecular evaluations of four patients from three unrelated families, including two previously reported siblings. Molecular testing comprised targeted next-generation sequencing or whole-exome sequencing, followed by Sanger sequencing for variant confirmation and familial segregation analysis where feasible. **Results**: Four *PLOD2* variants (NM_182943.3) were identified: homozygous c.1885A > G (p.Thr629Ala) in two siblings; c.8dup (p.(Cys4MetfsTer35)) and c.2222G > A (p.(Gly741Glu)) in one patient; and homozygous c.2027A > C (p.(Tyr676Ser)) in one infant. Three variants were previously unreported. Two missense variants remained classified as variants of uncertain significance, and the phase of the two heterozygous variants detected in one patient could not be established because a paternal sample was unavailable. Clinical severity was variable: age at first fracture ranged from 3 months to 4 years, and cumulative fracture burden ranged from 3 to multiple recurrent fractures. One 10-year-old patient had a severe OI-like phenotype without congenital contractures. Older patients showed additional axial and pelvic involvement, including craniovertebral junction abnormalities and acetabular protrusion. **Conclusions**: This case series broadens the range of clinical presentations observed in *PLOD2*-associated disease and indicates that severe bone fragility may occur in the absence of congenital contractures. These findings support inclusion of *PLOD2* in the differential diagnosis of patients with unexplained bone fragility and progressive skeletal deformities. Additional well-characterized cases and functional studies are needed to refine genotype–phenotype correlations and clarify the clinical significance of newly identified variants.

## 1. Introduction

Bruck syndrome (BS) is an ultra-rare autosomal recessive disorder with an estimated prevalence of less than 1:1,000,000 [1]. In the current nosology of genetic skeletal disorders, BS belongs to the group of disorders characterized by increased bone fragility together with osteogenesis imperfecta (OI) [2]. The characteristic features of the disorder include congenital contractures of the large joints and multiple fractures of the long bones beginning in infancy or early childhood [3,4]. Additional manifestations include postnatal short stature due to joint contractures, pterygia, severe limb deformities, and progressive scoliosis. Unlike classic forms of OI, patients with BS usually do not present with blue sclerae, dentinogenesis imperfecta, or hearing loss [3,5].

The phenotype was first delineated by Viljoen et al. in 1989, when they described five children from three unrelated families with symmetrical congenital contractures of the knees and ankles and fractures occurring after minimal trauma [6]. The term “Bruck syndrome” was proposed because of the clinical similarity to a case reported by Alfred Brooke in 1878 [6]. BS is currently divided into two clinically indistinguishable types, I and II (OMIM: 259450 and OMIM: 609220, respectively) [7].

BS type 1 is caused by biallelic variants in *FKBP10*, which encodes FK506-binding protein 10, a chaperone that interacts with lysyl hydroxylase 2 (LH2) and regulates its activity [8,9]. BS type 2 is caused by biallelic variants in *PLOD2*, which encodes LH2. Deficiency of the bone-specific telopeptide form of LH2 leads to reduced hydroxylysyl pyridinoline cross-links and abnormal bone collagen cross-linking [10,11].

According to the Human Gene Mutation Database (HGMD), 38 pathogenic variants in *PLOD2* have been reported to date, most of them being missense variants [12]. However, clear genotype–phenotype correlations in *PLOD2*-associated disease have not yet been established. Available data indicate marked clinical heterogeneity, including incomplete and atypical phenotypes [3,13,14]. For clarity, in the present manuscript we use the term BS type II when referring to the classical clinical entity and *PLOD2*-associated disease when referring more broadly to the underlying molecular spectrum. Homozygous nonsense or frameshift variants have been proposed to be prenatally lethal [14,15]. In this context, additional well-characterized clinical observations remain important for refining the phenotypic spectrum of the disorder and improving interpretation of newly identified *PLOD2* variants.

In the present study, we describe the clinical, radiological, and molecular findings in four patients from three unrelated families with biallelic *PLOD2* variants and phenotypes within the spectrum of *PLOD2*-associated disease, including both classical BS2 and atypical presentations. The cohort includes two siblings whom we reported previously in a separate publication [16], as well as two new unrelated cases. This expanded descriptive case series further delineates the clinical variability associated with *PLOD2* and adds three previously unreported sequence variants to the molecular spectrum of *PLOD2*-associated disease.

## 2. Materials and Methods

### 2.1. Clinical Evaluation

To establish the diagnosis, all patients underwent comprehensive evaluation, including pedigree analysis, clinical examination, molecular genetic testing, and radiological assessment of the affected skeletal regions. The extent of imaging was determined by the patient’s age, clinical presentation, and availability of archived studies. Targeted evaluation of the craniovertebral junction and signs of acetabular protrusion was performed when appropriate imaging was available and/or when clinically indicated. Because of the retrospective and descriptive nature of this case series, imaging was not fully standardized across all patients.

### 2.2. Molecular Genetic Testing

In Family 1, targeted next-generation sequencing was performed. Genomic DNA was extracted from whole blood using the DNeasy kit (Qiagen, Hilden, Germany) according to the manufacturer’s standard protocol. DNA and library concentrations were measured on a Qubit 2.0 fluorometer using Qubit BR and Qubit HS reagents (Life Technologies Corporation, Eugene, OR, USA) according to the manufacturer’s standard protocol. Library preparation was performed using a methodology based on multiplex polymerase chain reaction amplification of target DNA regions. Next-generation sequencing was carried out on an Ion Torrent S5 sequencer (Thermo Fisher Scientific, Waltham, MA, USA). Mean coverage of the target regions was at least ×80, and 90–94% of target regions were covered at ≥×20. Primary sequencing data were processed using the standard automated algorithm provided by Ion Torrent.

In Families 2 and 3, whole-exome sequencing (WES) was performed. Genomic DNA was extracted from peripheral blood leukocytes using the QIAamp DNA Blood Mini Kit (Qiagen, Hilden, Germany) according to the manufacturer’s protocol. The concentration of DNA, fragmented DNA after ultrasonic treatment, libraries, and the final pool was measured on a Qubit 2.0 device using Qubit BR and Qubit HS reagents (Life Technologies Corporation, Eugene, OR, USA) according to the standard protocol. The size distribution of DNA fragments after ultrasonic treatment, as well as of libraries and the final pool, was assessed on a TapeStation 4200 device (Agilent Technologies, Waldbronn, Germany) using the manufacturer’s reagents (High Sensitivity DNA D1000) according to the standard protocol. For sample preparation, selective capture of DNA regions corresponding to the coding regions of 19,396 genes was performed using the Illumina TruSeq^®^ Exome Kit (Illumina, San Diego, CA, USA) and the IDT xGen^®^ Exome Research Panel (Integrated DNA Technologies, Coralville, IA, USA). Average whole-exome coverage exceeded ×60; the percentage of target regions covered at ≥×10 was 99%; and coverage uniformity (uniformity Pct > 0.2 × mean) was 99%. Primary sequencing data were processed using the standard automated algorithm provided by Illumina for data analysis, available on BaseSpace.

### 2.3. Variant Interpretation and Validation

Population frequencies of the identified variants were assessed using the 1000 Genomes Project, ESP6500, and the Genome Aggregation Database (gnomAD). Clinical relevance of the selected variants was evaluated using the OMIM database and the HGMD^®^ Professional database, version 2022.1. Variant interpretation was performed in accordance with the ACMG/AMP standards and guidelines for the interpretation of sequence variants [17].

Validation of variants identified in the probands, as well as genotyping of siblings and parents, was carried out by direct automated Sanger sequencing according to the manufacturer’s protocol. In Family 1, Sanger sequencing was performed on an ABI Prism 3100 instrument (Applied Biosystems, Foster City, CA, USA). In Families 2 and 3, Sanger sequencing was performed on an ABI Prism 3500xl device (Applied Biosystems, Waltham, MA, USA). Primer sequences were selected according to the reference sequence of the target regions of *PLOD2* (NM_182943.3). In family 3, paternal DNA was unavailable; therefore, the phase of the two heterozygous variants could not be experimentally established.

### 2.4. Ethics Statement

Written informed consent for clinical evaluation, molecular genetic testing, and publication of anonymized data was obtained from the patients’ parents or legal guardians. The study was conducted in accordance with the Declaration of Helsinki and approved by the Institutional Review Board of the Research Center for Medical Genetics, Russia (protocol code: 10/1; approved on 8 November 2021).

## 3. Results

### 3.1. Molecular Findings

**Figure 1:**
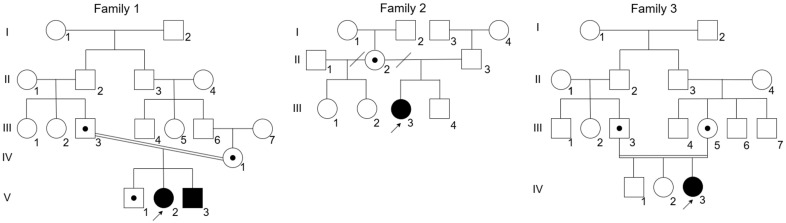
Pedigrees of the three families included in the current case series. Filled symbols indicate affected individuals, dots indicate heterozygous carriers, arrows indicate the probands, and double horizontal lines denote consanguinity. Roman numerals indicate generations, and Arabic numerals indicate individuals within each generation.

**Figure 2:**
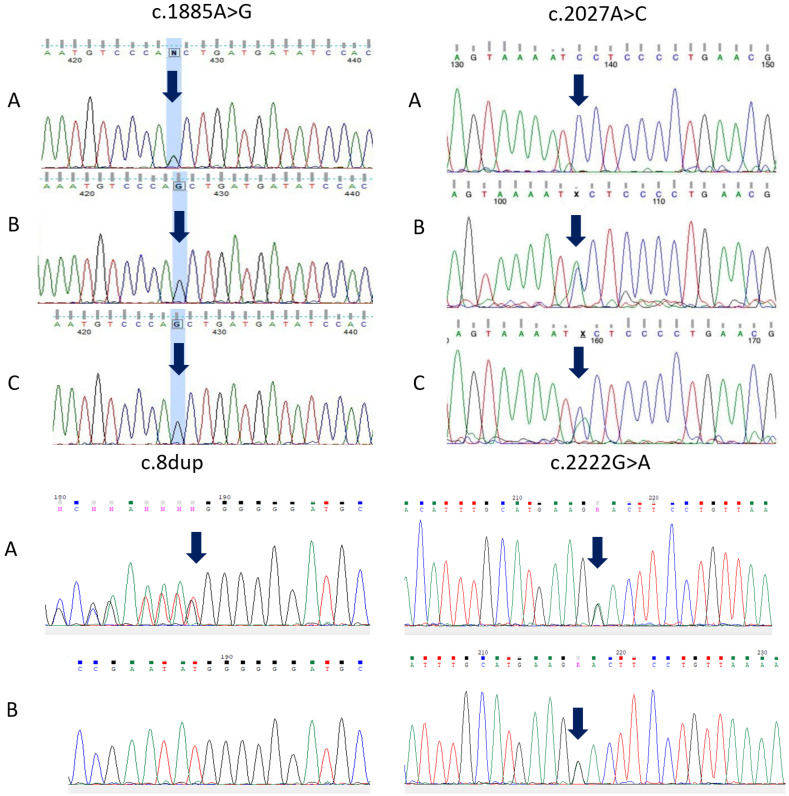
Sanger sequencing chromatograms of PCR products. Arrows indicate the positions of the identified *PLOD2* variants: c.1885A > G, c.2027A > C, c.8dup, and c.2222G > A, in the proband (A), mother (B), and father (C), where available; father not available for patient 3.

The study included four patients from three unrelated families, including two affected full siblings from Family 1 (Figure 1). Four nucleotide variants were identified in *PLOD2* (NM_182943.3): c.1885A > G (p.Thr629Ala), c.8dup (p.(Cys4MetfsTer35)), c.2222G > A (p.(Gly741Glu)), and c.2027A > C (p.(Tyr676Ser)) (Table 1). Three variants (c.8dup, c.2222G > A, and c.2027A > C) were previously unreported. According to ACMG/AMP criteria, c.8dup was classified as pathogenic, c.1885A > G as likely pathogenic, and c.2222G > A and c.2027A > C as variants of uncertain significance (VUS). Sanger sequencing confirmed the identified variants in the probands and supported segregation in the available family members (Figure 2). In family 3, paternal DNA was unavailable; therefore, the phase of the two heterozygous variants could not be experimentally established.

The missense variant c.1885A > G in exon 18 of *PLOD2*, previously reported by our group and resulting in substitution of threonine by alanine at position 629 (p.Thr629Ala), was identified in the homozygous state in two full siblings (P1 and P2) [16]. Homozygosity was confirmed by direct Sanger sequencing, and both parents were heterozygous carriers. The variant is located in a highly conserved region of *PLOD2* (phyloP100 = 8.042) and is present in gnomAD v3.1.2 at an extremely low frequency (0.000399%). A different homozygous change affecting the same codon, c.1886C > T (p.Thr629Ile), has previously been described in a patient with BS [10]. In addition, several nearby missense variants have been reported in BS, including p.Arg619His (c.1856G > A), p.Gly622Cys (c.1864G > T), and p.Gly622Val (c.1865G > T) [3,5]. These findings support the functional and evolutionary importance of this region of LH2 [10].

Patient 3 carried two previously unreported *PLOD2* variants: c.8dup (p.(Cys4MetfsTer35)) in exon 1 and c.2222G > A (p.(Gly741Glu)) in exon 20. The c.8dup variant is a frameshift predicted to introduce a premature termination codon and is therefore expected to result in nonsense-mediated mRNA decay or, if translated, a truncated protein. The missense variant c.2222G > A affects the C-terminal catalytic domain (2OG-Fe(II) oxygenase domain) and lies in a conserved region of the protein (phyloP100 = 7.48). Sanger sequencing showed that the mother was heterozygous for c.2222G > A, whereas c.8dup was absent in her DNA. Paternal DNA was unavailable for testing; therefore, although the two variants are likely to be in trans, this could not be confirmed experimentally, and the molecular findings in this case should be interpreted with appropriate caution.

In patient 4, a previously unreported missense variant, c.2027A > C (p.(Tyr676Ser)), in exon 19 of *PLOD2* was identified in the homozygous state. The p.(Tyr676Ser) substitution is located in the C-terminal catalytic domain of LH2, spanning amino acid residues 548–758 and containing the Fe^2+^-binding and 2-oxoglutarate-binding sites required for lysine hydroxylation [18]. The variant is absent from gnomAD v3.1.2. Based on ACMG criteria (PM2, PP3, PP2), it was classified as a variant of uncertain significance (VUS) (Table 1). Homozygosity was confirmed by direct Sanger sequencing, and both parents were heterozygous carriers. Thus, in this patient, the molecular findings are suggestive of *PLOD2*-associated disease but remain genetically inconclusive at the variant-classification level.

No direct functional or biochemical validation was available for the newly identified variants.

### 3.2. Clinical and Radiological Findings

The clinical presentation was markedly heterogeneous (Table 2). Age at first fracture ranged from 3 months to 4 years, and the cumulative fracture burden ranged from 3 to more than 70. Congenital contractures and foot deformities were present in patients 2 and 4, whereas patients 1 and 3 had no congenital contractures. Notably, patient 3 showed a severe osteogenesis imperfecta-like phenotype with pronounced long-bone deformities despite the absence of congenital contractures at 10 years of age.

Functional impairment was substantial in all patients but varied in severity. Patient 1 was able to walk with crutches, whereas patients 2 and 4 were able to sit but not walk, and patient 3 was unable to sit or walk independently. Spinal deformity was present in the three older patients.

Dedicated assessment of the craniovertebral junction and acetabular morphology was available for patients 1–3, but not for patient 4; therefore, these findings were recorded as not determined for patient 4 in Table 2. Among the three evaluated older patients, platybasia was present in all cases, basilar invagination in two, Chiari I malformation in one, and acetabular protrusion in all three. These findings indicate that axial and pelvic involvement may become clinically relevant during follow-up, although the present series is descriptive and too small to estimate their frequency.

Bone mineral density data were available for the two siblings from Family 1 and showed reduced lumbar spine Z-scores. Bisphosphonate therapy was initiated in the three older patients at 4, 6, and 10 years of age, respectively. Orthopedic interventions included femoral osteotomies with telescopic rodding in patients 1 and 2 and left Achilles tendon surgery in patient 4.

### 3.3. Brief Patient Descriptions

#### 3.3.1. Patient 1

**Figure 3:**
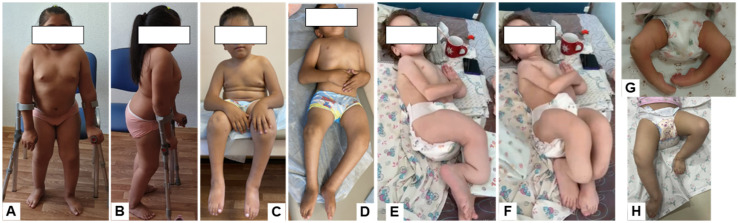
Clinical photographs of the examined patients: (**A**,**B**) Patient 1; (**C**,**D**) Patient 2; (**E**,**F**) Patient 3; (**G**,**H**) Patient 4.

**Figure 4:**
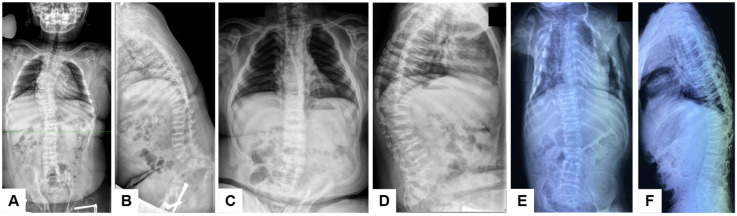
Anteroposterior and lateral radiographs of the spine in patients P1–P3. P1, 13 years (**A**,**B**): right-sided scoliosis of 40 degrees, kyphosis of 33 degrees, platyspondyly, and sacrococcygeal abnormalities. P2, 10 years (**C**,**D**): localized pathological thoracic kyphosis of 45 degrees with reduced vertebral body height and vertebral wedging, lumbar hyperlordosis, and a left-sided thoracolumbar scoliotic curve of 26 degrees. P3, 10 years (**E**,**F**): sequelae of multiple rib fractures with callus formation, pseudo-butterfly configuration of the biconcave vertebral bodies, S-shaped clavicular deformities, narrowing of the upper thorax, and asymmetric platyspondyly with anterior wedging and secondary kyphosis.

**Figure 5:**
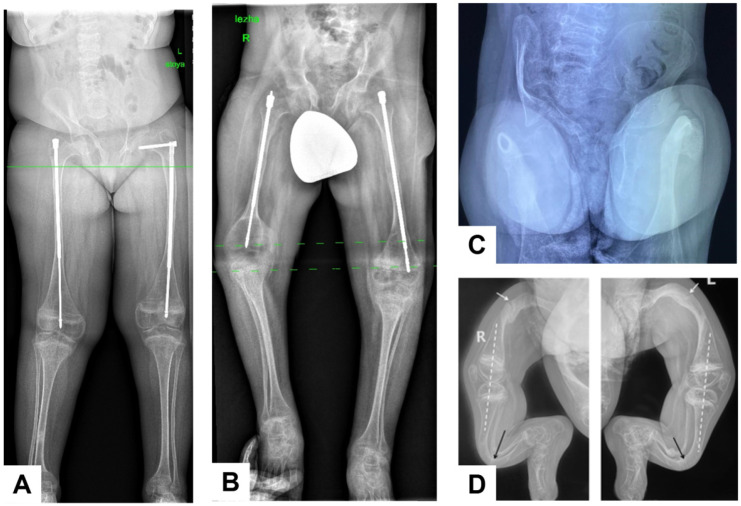
Radiographic findings of the pelvis and lower extremities in patients P1-P3. Common pelvic changes included narrowing of the anterior pelvic ring, protrusio acetabuli, and vertically oriented obturator foramina in all patients (**A**–**C**). Telescopic intramedullary rodding of the femora was present in P1 and P2 (**A**,**B**). In P3, the anteroposterior radiograph of the hips (**C**) additionally demonstrated asymmetric pelvic deformity, projected coxa vara, and marked osteoporosis of the proximal femora (white dotted lines). Radiographs of the lower extremities in P3 (**D**) showed multiplanar and multilevel deformities of the long bones, including procurvatum, varus, and rotational deformities of both femora with areas of pathological bone remodeling at the apices of the deformities (white arrows), procurvatum deformities of the tibial and fibular shafts with secondary thickening at the apices of the tibiae (black arrows), and preserved sagittal alignment of the knees (white dotted lines), consistent with full knee extension and absence of flexion contractures or juxta-articular deformities.

Patient 1 was a 13-year-old girl born to consanguineous parents. The disease manifested at 1 year of age with the development of flexion contractures of the knees and loss of independent ambulation; the first femoral fracture occurred at 3 years of age. At 4 years, spinal radiographs revealed compression fractures of the Th12–L1 vertebral bodies. At examination, her height deficit was −3.32 SD; she had thoracolumbar kyphoscoliosis, knee flexion contractures with an extension deficit of up to 20°, and ambulated with crutches (Figure 3A,B). Lumbar densitometry showed reduced bone mineral density (BMD) (Z-score down to −3.6); intermittent bisphosphonate therapy had been administered since 6 years of age. At 8 years, she underwent corrective femoral osteotomies with telescopic rodding. Spinal radiographs demonstrated a 33° kyphotic deformity at Th9–L2 and a 40° scoliotic deformity at Th9–L4; the sacrum was horizontal and the coccyx deviated by 90° (Figure 4A,B). Radiographs of the pelvis and lower extremities also showed narrowing of the anterior pelvic ring, protrusio acetabuli, and vertically oriented obturator foramina (Figure 5A). Magnetic resonance imaging of the craniovertebral region revealed Chiari I malformation.

#### 3.3.2. Patient 2

Patient 2 was a 10-year-old boy, the full brother of patient 1. From birth, he had flexion contractures of the elbows and knees (up to 90°), cubital and popliteal skin pterygia, and equinovalgus foot deformities, leading initially to a diagnosis of arthrogryposis. Staged correction included casting, orthotic management, and surgery on the feet and knees; the contractures recurred with growth. The first femoral fracture occurred at 3 years of age. At 4 years, lumbar densitometry demonstrated severe osteoporosis (Z-score −5.3), and intermittent bisphosphonate therapy was initiated. At 5 years, bilateral two-level corrective femoral osteotomies with telescopic rodding were performed (Figure 5B). At examination, height deficit was −3.2 SD; the patient was unable to walk independently. Contractures of the elbows, hips, and knees, as well as equinovalgus foot deformities, persisted (Figure 3C,D). Spinal radiographs showed platyspondyly, wedge deformities of the vertebral bodies, pathological thoracic kyphosis up to 45°, and a scoliotic curve of 26° (Figure 4C,D).

#### 3.3.3. Patient 3

Patient 3 was a 10-year-old girl born to non-consanguineous parents. She had a very severe OI-like phenotype with very early onset: the first fracture of the right femur occurred at 3 months of age, followed by a recurrent fracture at 7 months. From 1.5 years onward, fracture frequency increased, and by the age of 10 years the estimated cumulative fracture burden exceeded 70 episodes, based on available medical records and parental history. From the age of 3 years, progressive deformities of the long bones developed against a background of multiple fractures. She had never walked, and no joint contractures were present even at 10 years of age. At examination, she had marked short stature (−8.16 SD) due to multilevel deformities and shortening of the lower limb bones, muscle hypotrophy, and secondary lower thoracic kyphosis; the lower limbs were non-weight-bearing (Figure 3E,F). She could neither sit independently nor be verticalized or walk, moved mainly within the bed, and required assistance with self-care. Radiographs showed Wormian bones and open cranial sutures, platybasia and signs of basilar impression. Radiographs of the spine and thorax showed multiple rib fractures, mild platyspondyly, signs of previous vertebral compression fractures, and secondary kyphosis (Figure 4E,F). Radiographs of the pelvis and hips showed acetabular protrusion, asymmetric pelvic deformity, projected coxa vara, and marked osteoporosis of the proximal femora (Figure 5C). Multiple fractures and pronounced multiplanar deformities of the long bones of the extremities, with angulation exceeding 90°, marked cortical thinning, and disorganization of load-bearing trabecular lines, were also observed (Figure 5D).

#### 3.3.4. Patient 4

Patient 4 was a 1-year-5-month-old girl born to consanguineous parents. Congenital foot deformities and knee contractures were noted from birth, leading initially to a diagnosis of arthrogryposis. Beginning on day 7 of life, staged casting of the lower limbs was performed over a period of 3 months; at 1.5 months of age, surgery on the left Achilles tendon was performed. The first fracture, involving the bones of the left forearm, occurred at 6 months of age; at 1 year 2 months, she sustained a displaced fracture of the upper third of the right femur and a fracture of the upper third of the right tibia. At examination, height was −0.4 SD, psychomotor and speech development were age-appropriate, and she could sit independently but not walk. No scoliosis was detected. The upper limbs were without deformities and had preserved range of motion. In the lower limbs, flexion contractures of the knees and equino-cavo-adducted foot deformities were present, more pronounced on the left, with reduced weight-bearing capacity (Figure 3G,H).

## 4. Discussion

BS2 is an ultra-rare *PLOD2*-associated disorder traditionally regarded as an autosomal recessive form of osteogenesis imperfecta combining pronounced bone fragility with congenital joint contractures and, in some cases, pterygia [13,19]. However, the accumulation of molecularly confirmed observations clearly demonstrates that *PLOD2*-associated disorders form a phenotypic continuum in which classical BS2 occupies a central position within this spectrum. At one end of the spectrum are relatively mild OI-like phenotypes with preserved joint mobility, whereas at the other end are extremely severe forms, including perinatally lethal skeletal dysplasias with marked bowing of the long bones and a complicated intrauterine course. This concept has not only theoretical but also practical importance: when biallelic pathogenic variants in *PLOD2* are identified, the full range of possible phenotypes and reproductive risks, including the possibility of perinatally lethal forms, should be discussed with the family.

The present observations confirm marked inter- and intrafamilial variability of the *PLOD2*-associated phenotype. In our series, age at first fracture ranged from 3 months to 4 years, and the number of fractures ranged from a few to numerous; contractures were absent in some patients and varied in both age at onset and severity in others. However, any genotype–phenotype inferences remain tentative. The cohort is small, two novel missense variants remain classified as variants of uncertain significance, no direct biochemical or functional validation was available for the newly identified variants, and in patient P3 the phase of the two heterozygous variants could not be experimentally established because paternal DNA was unavailable. Accordingly, the molecular findings in this study are best interpreted as descriptive and hypothesis-generating rather than as evidence for stable genotype–phenotype rules.

One clinically important observation from the present series is that the absence of congenital contractures does not exclude *PLOD2*-associated disease. Patient P3 exhibited a severe OI-like phenotype with a very early fracture onset and marked long-bone deformities; however, congenital contractures were absent even at 10 years of age. Similar atypical presentations have also been reported previously, in which bone fragility dominated the clinical picture, whereas the joint component was minimal or absent [3,13,14,20]. A comparison of published molecularly confirmed cases of *PLOD2*-associated disease with explicitly documented absence of congenital contractures is provided in Supplementary Table S1. At the same time, the clinical observations currently available do not allow a reliable estimate of the frequency of *PLOD2*-associated phenotypes without contractures, and the pathogenic basis of this disease presentation remains undefined. From a practical perspective, *PLOD2*-associated disease should be considered in the differential diagnosis of patients with severe or unusual OI-like phenotypes, particularly in the presence of progressive deformities, scoliosis, or additional features related to contractures.

From a diagnostic standpoint, these observations also highlight the importance of the differential diagnosis. In patients with predominant bone fragility, deformity, and recurrent fractures, *PLOD2*-associated disease may overlap clinically with severe forms of osteogenesis imperfecta, including *FKBP10*-related disease (BS 1), because both disorders affect collagen cross-linking and may present with fractures, contractures, and progressive skeletal deformity [8,9]. In infants presenting primarily with congenital contractures or pterygia, arthrogryposis syndromes and other skeletal dysplasias may also enter the differential diagnosis. Likewise, some spinal and pelvic abnormalities described in our patients are not unique to *PLOD2*-associated disease and may also be encountered in severe OI. Therefore, these findings should be interpreted as supportive rather than disease-specific, and molecular testing remains essential for accurate diagnosis.

Intrafamilial variability in siblings P1 and P2 further highlights the diagnostic challenges of the early disease period. In P2, marked contractures and pterygia led to a diagnosis of arthrogryposis at birth, which shaped subsequent management and prompted early orthopedic interventions. In P1, knee contractures developed gradually beginning at 10 months of age, creating an atypical clinical picture for BS and requiring differential diagnosis with other hereditary skeletal disorders, including mucopolysaccharidosis and spondyloepiphyseal dysplasia. In both cases, the key clues to BS2 were the subsequent occurrence of fractures and the detection of osteoporosis. Thus, an arthrogryposis-like onset should be regarded as one of the typical clinical pitfalls of BS2, whereas the appearance of fractures and low BMD should prompt reconsideration of the diagnosis and expansion of molecular genetic testing.

In addition to fractures and contractures, our series suggests that craniovertebral junction and hip abnormalities may represent clinically relevant components of long-standing *PLOD2*-associated disease. In the older patients, imaging showed platybasia, signs of basilar impression, Chiari I malformation in one case, and acetabular protrusion in those with available hip radiographs. These findings should, however, be interpreted with caution, because targeted assessment of these regions was available mainly in the older patients. Moreover, similar craniovertebral and pelvic abnormalities may also occur in severe forms of OI. Therefore, our data do not establish these findings as specific markers of *PLOD2*-associated disease, but they do support considering focused surveillance of the craniovertebral junction and hips, especially in patients with severe or progressive deformity and in those undergoing orthopedic planning.

The clinical variability of *PLOD2*-associated conditions should be considered in the context of their molecular mechanism. *PLOD2* encodes LH2, which hydroxylates telopeptidyl lysine residues of type I collagen and thereby enables formation of mature bone-specific cross-links [10]. LH2 deficiency is associated with a reduced proportion of hydroxylysyl pyridinoline cross-links and the formation of a mechanically compromised collagen matrix, which pathogenetically explains the pronounced bone fragility [11,21]. The C-terminal catalytic domain is critical for enzyme function and harbors a substantial proportion of pathogenic missense variants; however, other types of changes, including frameshift, nonsense, and splice variants, have also been reported [15,22]. Several publications have suggested that homozygous nonsense and frameshift variants causing marked loss of protein function may be associated with a phenotype resembling kyphomelic or mesomelic dysplasia, including stillbirths and early infant death [14].

From the standpoint of molecular interpretation, the variants identified in our patients affect functionally important regions of the protein. In siblings P1 and P2, p.Thr629Ala is located in a critical region of the catalytic domain where pathogenic amino acid substitutions have previously been described [3,5]. Of particular interest, c.1885A > G (p.Thr629Ala), detected in the siblings, has recently been investigated in a dedicated molecular and cellular study [23]. In cell models (HEK293 and dermal fibroblasts), the mutant protein retained its typical localization in the endoplasmic reticulum, arguing against a disease mechanism based solely on gross intracellular trafficking defects. At the same time, expression of p.Thr629Ala was associated with measurable cellular effects, including reduced cell migration in a scratch-wound assay and transcriptomic changes in a patient-derived sample. Taken together, these findings further support the functional relevance of the substitution at the highly conserved Thr629 residue and its contribution to the BS2 phenotype [23].

In contrast, the molecular interpretation of patient P3 requires greater caution. This patient harbored a truncating variant together with a missense variant located in the C-terminal catalytic domain (2OG-Fe(II) oxygenase domain). In the context of a very severe but viable phenotype, this combination may be consistent with the hypothesis that some residual LH2 function can persist when at least one allele is not an obvious null variant. Published observations are broadly in line with this concept: the homozygous nonsense variant p.Trp561* was associated with death at 4 months of age [14]. In addition, Zhang et al. in 2021 described a family with multiple pregnancy losses and recurrent intrauterine skeletal dysplasia; prenatal evaluation showed marked limb shortening and bowing of the long bones, and the affected fetus carried compound heterozygous *PLOD2* variants c.2038C > T (p.Arg680*) and c.191_201+3delATACTGTGAAGGTA (p.Tyr64Cysfs*12), while immunohistochemistry demonstrated markedly reduced *PLOD2* expression in osteochondral tissue [15]. Experimental mouse models also support the biological relevance of complete loss of function, as global *PLOD2* knockout results in early embryonic lethality [24]. However, because paternal DNA was unavailable, the two variants identified in P3 should be described as presumed to be in trans rather than as proven compound heterozygous variants, and any discussion of a dose-dependent model or residual LH2 activity should therefore be considered preliminary. In addition, direct functional validation is lacking for the newly identified variants.

A similarly cautious interpretation is warranted for patient P4. The homozygous p.(Tyr676Ser) variant occurs in a consanguineous family, segregates with heterozygous carrier status in the parents, is absent from population databases, and affects the catalytic domain, making it a plausible candidate contributor to the phenotype. Nevertheless, under the current evidence framework it remains a VUS, and no direct functional or biochemical validation was available in this study. In ultra-rare disorders, reclassification often depends on the accumulation of comparable cases and on additional functional data, such as collagen cross-linking studies or assessment of LH2 activity and expression. Until such data become available, the relationship between p.(Tyr676Ser) and disease should be presented as strongly suggestive but not definitive.

## 5. Conclusions

Overall, this descriptive case series broadens the clinical and molecular observations associated with *PLOD2*-related disease while also underscoring the limits of inference from a very small cohort. Our data support considering *PLOD2* in the differential diagnosis of patients with unexplained bone fragility, progressive deformities, and arthrogryposis-like presentations, including some severe OI-like cases without congenital contractures. At the same time, larger series, standardized phenotyping, segregation data, and functional studies will be necessary before stronger conclusions can be drawn about phenotype boundaries, surveillance recommendations, or genotype–phenotype correlations.

## Figures and Tables

**Table 1:** *PLOD2* variants identified in the current case series.

Patient	cDNA Change	Protein Change	Exon	Zygosity	Novelty Status	Segregation/Phase Status	gnomAD v3.1.2	ACMG Classification
P1, P2 *	c.1885A > G	p.Thr629Ala	18	Hom	Previously reported [16]	Homozygous in both siblings; both parents were heterozygous carriers	0.000399%	Likely pathogenic (PM2, PM5, PP3, PM1, PP1-M)
P3	c.8dup	p.(Cys4MetfsTer35)	1	Het	Novel	Absent in mother; paternal DNA unavailable	0.001637%	Pathogenic (PM2, PVS1, PP5)
P3	c.2222G > A	p.(Gly741Glu)	20	Het	Novel	Maternally inherited; paternal DNA unavailable; phase not experimentally established	Absent from gnomAD	VUS (PM2, PP3, PM1)
P4	c.2027A > C	p.(Tyr676Ser)	19	Hom	Novel	Homozygous in proband; both parents were heterozygous carriers	Absent from gnomAD	VUS (PM2, PP3, PP2)

* P1 and P2 are full siblings. Abbreviations: ACMG—American College of Medical Genetics; gnomAD—Genome Aggregation Database; VUS—variant of uncertain significance.

**Table 2:** Clinical, functional, and radiological characteristics of the patients.

Characteristic	Patient 1	Patient 2	Patient 3	Patient 4
Consanguinity	+	+	−	+
Ethnicity	Tajik	Tajik	Russian	Uzbek
Age (years)	13	10	10	1 year 5 months
Sex	female	male	female	female
Congenital contractures	−	+	−	+
Pterygia	−	+	−	−
Foot deformities	−	+	−	+
Age at first fracture	3 years	3 years	3 months	6 months
Number of fractures (*n*)	6	3	>70, estimated	3
Height (SDS)	−3.3	−3.2	−8.2	−0.4
Mobility	walks with crutches	sits, does not walk	does not sit, does not walk	sits, does not walk
Spinal deformity	kyphoscoliosis	kyphoscoliosis	thoracolumbar kyphosis	none
Craniovertebral abnormalities	platybasia; basilar invagination; Chiari I	platybasia	platybasia; basilar invagination	ND
Acetabular protrusion	+	+	+	ND
BMD Z-score (L1–L4)	−0.7 to −3.6	−2.5 to −5.3	ND	ND
Bisphosphonate therapy (age at initiation)	6 years	4 years	10 years	—
Orthopedic surgeries (key)	femoral osteotomies + telescopic rods (8 years)	femoral osteotomies + telescopic rods (5 years)	—	left Achilles tendon surgery (1.5 months)

BMD, bone mineral density; ND, not determined.

## Data Availability

The original contributions presented in this study are included in the article/Supplementary Materials. Further inquiries can be directed to the corresponding author.

## Supplementary Materials

The following supporting information can be downloaded at: https://www.mdpi.com/article/10.3390/genes17050556/s1, Table S1: Published molecularly confirmed cases of *PLOD2*-associated disease with explicitly documented absence of congenital contractures [3,13,14,20].
