# Supplementary material for: Clinical Variability Within the PLOD2-Associated Phenotypic Continuum: Three Novel Variants in Four Patients from a Descriptive Case Series
Source: Genes (Basel). 2026 May 5;17(5):556. doi: 10.3390/genes17050556 (PMC13206548; doi:10.3390/genes17050556)
Supplement: Supplementary file 1 [file genes-17-00556-s001.zip › genes-4289618-supplementary.pdf]

Supplementary Table S1. Molecularly confirmed PLOD2-associated cases with explicit absence of congenital contractures in the literature and in the present series.

Only literature cases with explicit documentation of absent congenital contractures were included.

Study	Informative case(s)	PLOD2 variant(s)	Contracture status	Fracture onset / burden	Selected phenotype
Puig-Hervás et al., 2012	Family 7 proband (15-year-old male)	Homozygous c.1358+5G>A	No congenital or postnatal contractures	Fractures from 2 y; ~10 fractures/year	Severe AR-OI phenotype with kyphoscoliosis, Wormian bones, osteopenia/borderline osteoporosis, generalized hypotonia, and no pterygia.
Puig-Hervás et al., 2012	Family 8, patient II-2 (18-year-old male)	Compound heterozygous c.1864G>T (p.Gly622Cys) + c.2122-2A>G	No congenital contractures; normal joints	2 tibial fractures during the first year of life	Mild OI phenotype with pes valgus, slight bowing of the legs, very mild scoliosis, borderline lumbar osteoporosis, and normal ambulation.
Caparrós-Martín et al., 2017	Family 91 proband (9-year-old girl)	Homozygous c.1358+5G>A	No congenital contractures	First fracture at 6 y; 1–2 fractures/year thereafter	Short stature, blue sclerae, very mild hypotonia, Wormian bones, osteoporosis, and mild kyphosis.
Leal et al., 2018	Patient 4 (male with moderate OI)	Homozygous c.1764G>T (p.Trp588Cys)	No joint contractures (normal joints)	Fractures from 2 y; total 5 fractures, including tibial insufficiency/stress fractures	Moderate OI with low lumbar BMD, severe bone pain, recurrent tibial insufficiency fractures, and no scoliosis.
Mumm et al., 2020	Young woman	Compound heterozygous c.797G>T (p.Gly266Val) + c.1280A>G (p.Asn427Ser)	Without congenital joint contractures or pterygia	Fragility fractures from ~3 y; recurrent poorly healing fractures	OI-like phenotype with blue sclerae, osteopenia, Wormian bones, progressive scoliosis, protrusio acetabuli, short stature, and additional dysmorphic features.
Present series	Patient 1	Homozygous c.1885A>G (p.Thr629Ala)	No congenital contractures; knee flexion contractures developed during the first year of life	First fracture at 3 y; 6 fractures	Later-onset joint involvement with kyphoscoliosis, Chiari I malformation, and acetabular protrusion.
Present series	Patient 3	c.8dup (p.Cys4MetfsTer35) + c.2222G>A (p.Gly741Glu); phase not experimentally established	No congenital contractures; no joint contractures even at 10 y	First fracture at 3 mo; estimated cumulative fracture burden >70 by age 10 y	Very severe OI-like phenotype with pronounced multiplanar long-bone deformities, platybasia/basilar impression, and acetabular protrusion.

Abbreviations: AR-OI, autosomal-recessive osteogenesis imperfecta; BMD, bone mineral density; OI, osteogenesis imperfecta.
